# Rapid virological&End treatment response of patients treated with Sofosbuvir in Chronic Hepatitis C

**DOI:** 10.12669/pjms.334.12785

**Published:** 2017

**Authors:** Muhammad Shoaib Siddique, Sana Shoaib, Alvia Saad, Hamna Javed Iqbal, Noureen Durrani

**Affiliations:** 1 Dr. Muhammad ShoaibSiddique, FCPS Gastroenterology. Consultant Gastroenterologists, Memon Medical Institute & Hospital, Karachi, Pakistan; 2 Dr. Sana Shoaib, FCPS Medicine. Consultant Medicine, Memon Medical Institute & Hospital, Karachi, Pakistan; 3 Dr. AlviaSaad, FCPS Registrar General Medicine. Incharge Emergency, Memon Medical Institute & Hospital, Karachi, Pakistan; 4 HamnaJavedIqbal, Student, Research Coordinator, Memon Medical Hospital, Karachi, Memon Medical Institute & Hospital, Karachi, Pakistan; 5 NoureenDurrani, Statistician & Researcher, Memon Medical Institute & Hospital, Karachi, Pakistan

**Keywords:** Sofosbuvir, Hepatitis C virus

## Abstract

**Objective:**

To determineRapid & End treatment response of patients treated with Sofosbuvir in Chronic Hepatitis C at tertiary care hospital.

**Methods:**

It was an observational study conducted at Memon Medical Institute from January 2016 to July 2017. The inclusion criteria for patients was 18 years of age or older, having chronic infection with HCV. Total=201 received sofosbuvir with or without interferon in our OPDs. Patients were categorized into Treatment naïve, treatment experienced and decompensated chronic liver disease. Pregnant patients and those not willing to participate were excluded. Initially genotyping and Quantitative HCV RNA test was done.

**Results:**

A total of 201 subjects were included in the study with mean age of the patients was 46.22± 14.41 years. Of 201 patients, n= 131 (65.2%) chronic hepatitis C, compensated cirrhosis n= 47(23.4%), and with decompensated cirrhosis n=23(11.4%). Most commonly genotype 3 n= 180 (89.6%) was present followed by genotype 1 n=9(4.5%), genotype 2 n=1(0.5%), genotype 4 n=1(0.5%). Of patients with genotype 3, 123 received dual therapy and 57 were given triple therapy. After one month of therapy HCV RNA by PCR, 200(99.5%) achieved RVR, 199(99%) achieved ETR and SVR achieved in 178(88.5%) while remaining 1 patient did not achieved RVR, 2 ETR and 12 patients did not achieved SVR and remaining 11 SVR lost follow up.

**Conclusion:**

Sofosbuvir has shown to be very effective andsuccessfulwith achievement of virological response with little or no resistance in all genotypes mainly genotype 3 treated in our study population. The promising results of our study will aid in better outcomes and therefore help in eradication of the virus.

## INTRODUCTION

Hepatitis C infection represents a global health problems affecting 200 million subjects worldwide.^1^-^3^ Previous Studies indicate that around, three to four million people are newly infected each year resulting in an estimated 350,000 deaths annually.^4^,^5^ Pakistan is estimated to have the second highest patient burden. Hepatitis C and the population suffering from this disease is said to be between 4.5-8.2%,^6^ among these 78% of subjects belong to genotype 3 in contrast to the western population where this genotype is less common.^7^

In the recent past the standard of treatment for chronic hepatitis C included pegylated interferon and ribavirin. However, a large number of subjects with HCV infection remained untreated due to absolute or relative contraindications to interferon therapy, like hepatic decompensation, autoimmune disease, and psychiatric illness.^8^ Also adverse effects like ‘flu-like symptoms, fatigue, fever, leucopenia or thrombocytopenia can be unpleasant and sometimes serious, leading to dose reduction or discontinuation of treatment.^9^ In this regard the introduction of Sofosbuvir has proven to be groundbreaking as far as cure for hepatitis C is concerned. Sofosbuvir is an oral nucleotide analogue inhibitor of the HCV-specific NS5B polymerase with in vitro activity against all HCV genotypes^10^ particularly genotype 1, 2, 3 and 4. The safety and efficacy of sofosbuvir as proved by ELECTRON trial previously has it the basis of combination antiviral therapy in patients with chronic HCV genotype 1, 2 and 3 infections, including both treatment-naive and treatment-experienced patients.^11^,^12^ No dose adjustment is required for creatinine clearance higher than 30 ml/minutes, along with minimal side effects and the advantage of once daily oral dose makes it superior and more desirable other therapies of HCV.^13^

According to international guidelines of EASL the response to therapy with sofosbuvir is judged initially by the rapid virological response (RVR) that is the undetectable viral load on PCR done at one month while the ultimate goal is to get a sustained virological response (SVR) done after 12 weeks of completion of therapy.^14^ International studies have shown that twelve weeks of treatment with Sofosbuvir in combination with peginterferon and ribavirin resulted in substantial decreases in the level of HCV RNA during therapy, leading to a sustained virologic response at 24 weeks after treatment in 92% of patients with HCV genotype 2 or 3 infection.^15^ The same regimen of 12 weeks of treatment with sofosbuvir, peginterferon and ribavirin resulted in a sustained virologic response 24 weeks after treatment in 89% of patients with HCV genotype 1 infection^16^ But all these statistics reflect the response rates in western population where the distribution of genotypes is different from that of Pakistani population.^17^-^19^ The treatment response of oral regimens of 80—85% have been seen while in genotype 3, response is not as good. So we may use RVR and EVR to modify treatment in our patients of genotype 3 to save cost and get better results.

Therefore the aim of this study was to determine the rapid and end treatment response of sofosbuvir based regime in Pakistani population.

## METHODS

It was an observational study, conducted at Memon Medical Institute from January 2016 to July 2017. For this study, data of HCV subjects attending outpatients (OPD) was obtained from computerized hospital management system (HMS). Variables that were obtained through administrative data included risk factors of Hepatitis C along with its chronic complications. Total number of patients n=201 have received sofosbuvir with or without interferon in our OPDs. Patients were categorized into Treatment naïve, Relapsers and Non responders. Among these categories patients were divided as Non cirrhotic and Cirrhotic furthermore, cirrhotic involves Compensated CLD and Decompensated CLD.

The inclusion criteria for patients was 18 years of age or older, having chronic infection with HCV. Patients included were treatment naïve, non responders or relapses’ and among these categories both cirrhotic and non-cirrhotic were included. The exclusion criteria included those that did not give informed consent and pregnant patients. Initially genotyping and Quantitative PCR test were done. It was planned to perform 4 PCRs during therapy for monitoring. One at the start of treatment, then at 4 weeks that is RVR, then at the end of treatment either 12 or 24 weeks depending on the type of therapy to see ETR and then 12 weeks after completion of therapy to see SVR.

After the initial PCR became positive, written informed consent was taken. Patients were given dual and triple therapy according to genotype as mentioned in European association for the study of liver (EASL) guidelines 20. Patient who were relapsers and non responders, and genotype 1, 4, 5, 6 were mainly given triple therapy while treatment naïve and cirrhotic were given dual therapy.

Dual therapy comprises of sofosbuvir 4oomg once daily and weight based ribavirin (1000mg daily for <75 kg and 1200mg for >75 kg in two divided doses) and in triple therapy pegylated interferon were added to this regime. Labs were repeated fortnightly to check for occurrence of any complications or side effects like anemia. The study was conducted in accord with the ethical principles that originated in the Declaration of Helsinki, after the study protocol is approved by the institutional review board.

## RESULTS

**Fig. 1:**
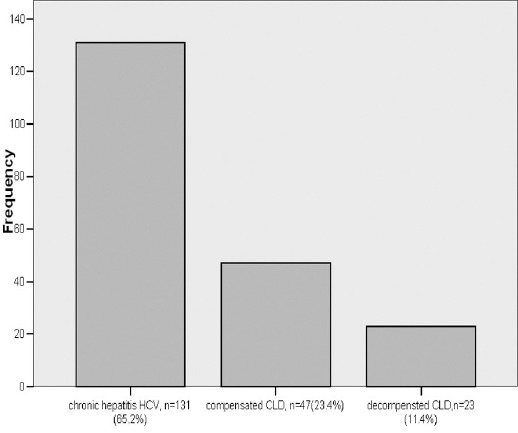
Cirrhosis.

A total of 201 subjects were included in this study the demographic and clinical characteristics of the patients at baseline are shown in Table-I. A total of 36 females had compensated cirrhosis while 90 were non cirrhotic chronic hepatitis C and 16 had decompensated cirrhosis (Fig. 1). There were 41 men with chronic hepatitis C, 11 compensated CLD and seven decompensated cirrhosis.

**Table-I:** Demographic Data.

*Demographic variables*	*Frequency (percentages) n=201*
***Age in years***	
Mean SD	46.22±14.41
***Gender***	
Female	59(29.4%)
Male	142(70.6%)

**Fig. 2:**
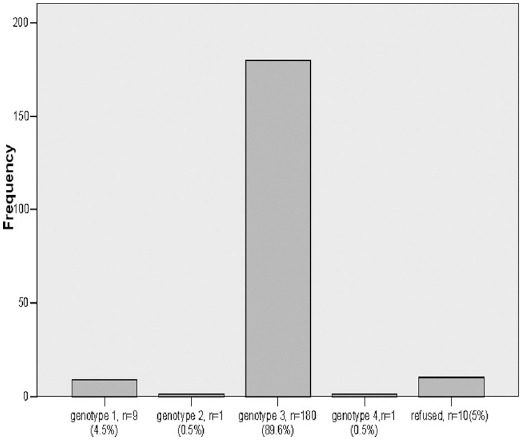
Genotype.

The co morbidities, among 201 patients, 7.5% women were diabetic and 92.5% were not diabetic, 11.1% male were having diabetes and 88.9% were non diabetic. Furthermore, 7.5% women were hypertensive and 92.5% were not and only 3.7% men were hypertensive and 96.3% were not. Neither women nor men had any ischemic heart disease. There were treatment naïve 133(66.2%) treatment experienced 45(22.4%) and decompensated CLD 23(11.4%). The genotypes frequency is shown in (Fig. 2)

In genotype 3 there were n=123 patients with dual therapy, n=57 of genotype 3 with triple therapy, in genotype 1 n=3 were on dual therapy and n=6 were on triple therapy, genotype 2 and 4 n=1 in both were on triple therapy. Patients refused for genotyping were 4 in triple therapy, and 6 patients refused from dual therapy. After one month of therapy HCV RNA by PCR of out of 201 patients, 200(99.5%) achieved RVR, only n=1(0.5%) case did not achieved RVR and treatment was stopped. Of 201, 199 (99%) ETR was achieved and in only n=2(1.0%) patients did not achieved ETR. The SVR of the study have also been achieved in 178 (88.5%) patients while n=12 did not achieve SVR and remaining n=11 lost follow up. All patients who were treated with dual therapy n=78 achieved RVR and n=40 with triple therapy achieved RVR. When results of those RVR achieved was compared with their ETR and SVR remarkable results were achieved.

## DISCUSSION

Pakistan is said to have the highest prevalence of hepatitis C and the population suffering from this disease is said to be between 4.5-8.2%.^6^,^21^-^24^ Ever since the introduction of a new oral drug ‘Sofosbuvir’ the cure rates for chronic Hepatitis C has reached dramatic heights. Sofosbuvir is an oral nucleotide analogue inhibitor of the HCV-specific NS5B polymerase with in vitro activity against all HCV genotypes.^10^-^12^ Pakistan has a predominance of genotype 3 which according to some studies is said to be around 78%.^21^-^23^ Sofosbuvir has a number of ideal properties, including pan genotypic activity, once daily dosing, no meal restrictions, few adverse effects, minimal drug-drug interactions, high genetic barrier to resistance, good safety and efficacy in patients with advanced liver disease, and excellent sustained virological response rates in patients with unfavorable baseline characteristics.

This study has found increased incidence of hepatitis C among age group of 46 years with male predominance. Studies have also shown varying incidence in gender and age groups. Tahir et al has found 30-59 yrs.’ of age and increased incidence among females 55%.^22^ Sirhindi et al has found increased among males.^24^ Our study also revealed increased frequency of genotype 3 compared to other genotypes. These results are similar to studies conducted in our study.

Our observational study done on 201 patients showed that the initial response of sofosbuvir regime given either as dual or triple therapy had excellent response rates and almost all patients enrolled had achieved RVR at one month of therapy. Among treatment Naïve and treatment experienced relapsers and decompensated CLD responded to both dual and triple therapy. Patients treated with Sofosbuvir in our study have shown excellent results with 99.5% achievement of RVR, 99% ETR of patients treated and SVR 98.5%. Studies have shown higher rates of SVR in patients treated with interferon free combinations in genotypes 1 and 2 especially with very good safety profile and favorable outcomes and without resistance in both cirrhotics and non-cirrhotics. Recently newer interferon free regimes have replaced chronic HCV management.^25^ Lawaitz et al has found trial of sofosbuvir in patients with moderate hepatic dysfunction due to chronic HCV and found good tolerability and good results with declining HCV RNA in 7 days of dosing.^26^ Sarwar et al in his observational study has also found 83.1% achieving SVR of which 89.2% achieving with triple therapy and 82.2% achieving with sofosbuvir/ribavirin therapy.^27^ The PROTON randomized double blind trial has also demonstrated sofosbuvir to be highly efficacious especially in genotypes 1-3 with achievement of SVR in greater than 90%.

Another large multicenter study HEATS has found 98.2% patients achieving RVR in HCV infected patients with well tolerability.^28^ The results of our study have found sofosbuvir to achieve 100% results of RVR and ETR in patients whose results have been collected and also SVR of patients chased also found 100% in both patients treated with dual and triple therapy.

## CONCLUSION

Sofosbuvir has shown to be very effective and successful achievement of virological response with little or no resistance in all genotypes mainly genotype 3 treated in our study population. Better outcomes are expected with promising results of our study.

### Authors Contribution

**MSS** envisioned, designed and collection of data with manuscript writing.

**SS and HJI** did data collection and also did manuscript writing.

**AS** proof reading of manuscript and takes responsibility as well as is answerable for all aspects of work related to precision and genuineness in all the work.

**ND** statistical analysis and its related aspect throughout research.
